# Use of digital self-care solutions for diabetes long-term management: a scoping review protocol

**DOI:** 10.1136/bmjopen-2025-100506

**Published:** 2025-10-29

**Authors:** Jorge César Correia, Cosette Fakih El Khoury, Dayana El Chaar, Stella Arakelyan, Alon Rasooly, Giulia Loffreda, Surabhi Joshi, Jean-David Cohen, Vincent De Andrade, Benoît Pétré, Luis Velez Lapão, Caroline Perrin, Zoltan Pataky

**Affiliations:** 1 Unit of Therapeutic Patient Education, WHO Collaborating Centre, Geneva University Hospitals, Geneva, Switzerland; 2 Faculty Diabetes Centre, Faculty of Medicine, University of Geneva, Geneva, Switzerland; 3 National Institute of Public Health Clinical Epidemiology and Toxicology, Beirut, Beirut Governorate, Lebanon; 4 Department of Nutritional Sciences, University of Toronto, Toronto, Ontario, Canada; 5 The University of Edinburgh Usher Institute of Population Health Sciences and Informatics, Edinburgh, UK; 6 Division of Tropical and Humanitarian Medicine, University of Geneva, Geneva, Switzerland; 7 Alliance for Health Policy and Systems Research (HSR), Science Division, World Health Organization, Geneva, Switzerland; 8 Department of Noncommunicable Diseases, Rehabilitation and Disability (NCD), World Health Organization, Geneva, Switzerland; 9 Rheumatology, University of Montpellier, Montpellier, France; 10 UFR SMBH, Education and Health Promotion Laboratory UR-3412, Sorbonne North Paris University, Villetaneuse, Île-de-France, France; 11 Graduate School of Nursing Sciences in Health Promotion, Sorbonne North Paris University, Villetaneuse, Île-de-France, France; 12 Public Health Department, Liege University, Liege, Belgium; 13 IDeaS Laboratory, UNIDEMI, NOVA University Lisbon NOVA School of Science and Technology, Caparica, Setubal, Portugal; 14 WHO Collaborating Centre on Health Workforce Policy and Planning, IHMT, Universidade Nova de Lisboa, Lisbon, Portugal; 15 Geneva Digital Health Hub, Faculty of Medicine, University of Geneva, Geneva, Switzerland

**Keywords:** General diabetes, Digital Technology, eHealth, Self-Management

## Abstract

**Abstract:**

**Introduction:**

Diabetes mellitus is a significant global health challenge, requiring innovative strategies to improve management and mitigate complications. Digital health technologies offer promising solutions to enhance diabetes self-care by providing real-time feedback, improving communication and supporting data-driven decision-making. Despite the increasing adoption of digital self-care interventions, there is a lack of comprehensive synthesis of evidence on their impact, accessibility and integration into healthcare systems. This scoping review aims to map existing research on digital self-care solutions for diabetes management, identify knowledge gaps and highlight best practices and key factors influencing adoption.

**Methods and analysis:**

The review will follow Arksey and O’Malley’s framework and adhere to Preferred Reporting Items for Systematic Reviews and Meta-Analyses Extension for Scoping Reviews guidelines. A systematic search will be conducted in Medline, Scopus, Embase, CINAHL and Google Scholar, focusing on studies published from January 2004 to December 2024 in English, French, Arabic, Portuguese, Spanish, Italian, Czech, Slovak and Chinese. Studies reporting on digital self-care solutions for diabetes management will be included, covering experimental and quasi-experimental study designs. Data extraction will cover study and participant characteristics, digital solution features, and barriers and facilitators to adoption. Ethical and equity considerations will also be analysed using established frameworks. Two reviewers will independently screen studies, with discrepancies resolved by a third reviewer.

**Ethics and dissemination:**

This scoping review will provide a comprehensive understanding of digital self-care solutions for diabetes management, offering insights to inform future research and enhance self-care practices globally. Findings will be disseminated through peer-reviewed publications, conferences and interest holder engagements to inform clinical practice and policy development. As this study involves the review of existing literature, ethical approval is not required.

**STRENGTHS AND LIMITATIONS OF THIS STUDY**

- This scoping review applies a rigorous and comprehensive search strategy across multiple databases and grey literature sources.
- It includes a wide range of study designs and real-world interventions to ensure diverse perspectives.
- Data extraction is aligned with established frameworks (eg, WHO Self-Care Framework, Digital Health Equity Framework).
- The review incorporates ethical and equity dimensions, including barriers in low-resource settings and artificial intelligence-driven interventions.
- The quality of included studies will not be assessed, as this is beyond the scope of a scoping review.

## Introduction

 Diabetes mellitus represents a major global health challenge, affecting millions of individuals worldwide and posing a substantial burden on health systems.^1^ As the prevalence of diabetes continues to rise, there is an urgent need for evidence-based innovative strategies to improve its management and mitigate associated complications.^2^ Among these strategies, therapeutic patient education (TPE) has emerged as a cornerstone of diabetes management,^3^ recognised for providing individuals living with diabetes the essential tools to better understand their condition, adhere to treatment plans and make informed lifestyle changes.^3 4^

In this context, digital health technologies and services represent promising tools for enhancing diabetes care management. These tools, when properly developed, offer opportunities to optimise self-management, strengthen communication between patients and healthcare providers, and support data-driven decision-making.^4^,^6^

The scope of digital technologies implementation in healthcare is vast. According to the WHO classification of digital health interventions, these technologies fall into several key categories, including interventions targeted at clients (eg, telemedicine and mHealth applications), healthcare providers (eg, decision support systems and electronic medical records) and health system or resource managers (eg, supply chain management and health workforce management tools).^7^ When properly aligned with care processes, they can provide real-time feedback, tailored recommendations and continuous monitoring, all of which are crucial for effective diabetes management.^8^ Additionally, they enable remote consultations and patient engagement, and ongoing support, reducing the need for frequent in-person visits and improving healthcare accessibility.^9^ Artificial intelligence (AI)-driven solutions, in particular, have gained traction in diabetes self-management by offering enhanced data analysis, automation and personalised interventions. These technologies have the potential to improve health outcomes by providing real-time insights and adaptive feedback based on individual user data.^10^

Self-care has emerged as a pivotal focus in diabetes management, emphasising the need to empower individuals to take control of their conditions. According to the WHO definition of self-care, it is ‘the ability of individuals, families and communities to promote health, prevent disease, maintain health and cope with illness and disability with or without the support of a health worker’.^11^ Self-care not only enhances health outcomes but also significantly improves quality of life.^12 13^

The WHO self-care intervention framework provides a structured approach to support individuals across various levels of healthcare engagement.^14^ Key self-care practices for individuals with diabetes (or for supporting carers) include monitoring blood glucose levels, adhering to medication regimens, maintaining a healthy diet and engaging in regular physical activity, as well as feeling comfortable about it.^15^

Given the heterogeneity of digital self-care solutions, outcomes assessed, and population groups targeted, a scoping review is appropriate to map the breadth of existing literature, identify key concepts and gaps, and inform future systematic reviews or meta-analyses. While several prior reviews have addressed digital interventions for diabetes,^1216^,^19^ these have typically focused on specific technologies, limited populations or lacked a self-care or equity perspective. This review aims to fill these gaps by applying a broader and more inclusive lens—covering multiple types of diabetes, real-world and AI-driven tools, WHO-aligned self-care classification, and digital health equity considerations across diverse settings. Furthermore, this scoping review seeks to better understand the key drivers and barriers to adoption, which will provide valuable insights into how these technologies can be optimised to support self-care in diverse populations. By identifying trends, challenges and opportunities in the field, this review will contribute to the development of evidence-based strategies that enhance diabetes self-management and ultimately improve patient outcomes.

## Methods

This scoping review will be conducted in accordance with the methodology framework proposed by Arksey and O’Malley,^20^ which involves five key stages: identifying the research question, identifying relevant studies, study selection, charting the data, and collating, summarising and reporting the results. This scoping review will adhere to the Preferred Reporting Items for Systematic Reviews and Meta-Analyses Extension for Scoping Reviews guidelines.^21^

### Stage 1: identifying the research question

The objective will focus on answering the following research question: what digital self-care systems for diabetes self-management have been developed and proposed for people living with diabetes?

This overarching question will help capture the range of digital health technologies and services used to support diabetes self-management, including mobile applications, wearable devices, telehealth services and other digital tools. The sub-questions of this review will be as follows:

- What types of digital self-care solutions are available for diabetes management?
- What are the usability aspects of these solutions, including acceptability, barriers and drivers?
- How do these solutions impact diabetes self-management and patient-provider relationships?
- What gaps exist in the current literature, and what directions should future research take?
- How accessible are these solutions to diverse populations, including those in low-resource settings, with varying levels of digital literacy or with disabilities?

Data collection is scheduled to begin in July 2025 and conclude by December 2025.

### Stage 2: identifying relevant studies

A systematic search will be conducted across the following databases: Medline (PubMed interface, 1946 onwards), Scopus (Elsevier interface, 1974 onwards), Embase (Elsevier interface, 1974 onwards) and CINAHL (EBSCOhost platform, 1981 onwards).

Grey literature will be identified using Google Scholar. Studies recommend screening the first 300 search results in Google Scholar, sorted by relevance, when conducting systematic reviews, which will guide our screening range to ensure comprehensive coverage of relevant literature.^22^ Inclusion will be limited to documents presenting empirical data or evaluations of digital self-care interventions (eg, government reports, non-commercial white papers, technical documentation from reputable institutions).

The search will focus on papers published from January 2004 to December 2024. The decision to restrict the timeframe to studies published from January 2004 to December 2024 ensures the inclusion of contemporary and relevant evidence that reflects the current state of digital self-care interventions.^23^ This period captures the era of rapid growth in digital health—marked by the emergence of smartphones, mHealth apps and AI-driven tools—and aligns with increased global momentum for digital health, including key policy frameworks introduced by the WHO. By focusing on this timeframe, the review aims to generate findings that are most applicable to current and future healthcare practices, while excluding outdated information from earlier technological paradigms.^23^

No limits will be applied to the searches apart from the language limitation, which will include studies published in English, French, Arabic, Portuguese, Spanish, Italian, Czech, Slovak and Chinese. These languages have been selected based on the linguistic proficiency of the research team, enabling comprehensive screening, data extraction and analysis without the need for translation services. This approach ensures a broader inclusion of relevant literature from diverse geographical regions while maintaining the accuracy and reliability of the review process.

Search terms and Boolean operators were iteratively developed and refined in consultation with a research librarian (VDA) to ensure a robust and targeted approach. A detailed PubMed search strategy is provided in online supplemental file 1 and will be adapted appropriately for other databases using relevant subject headings and platform-specific syntax.

In the event of an unmanageably large number of eligible studies, we will implement a staged prioritisation strategy based on population (eg, type 2 diabetes), intervention focus (eg, tools in active clinical use), and study design, while transparently documenting excluded studies and reasons.

### Stage 3: study selection

**Figure 1:**
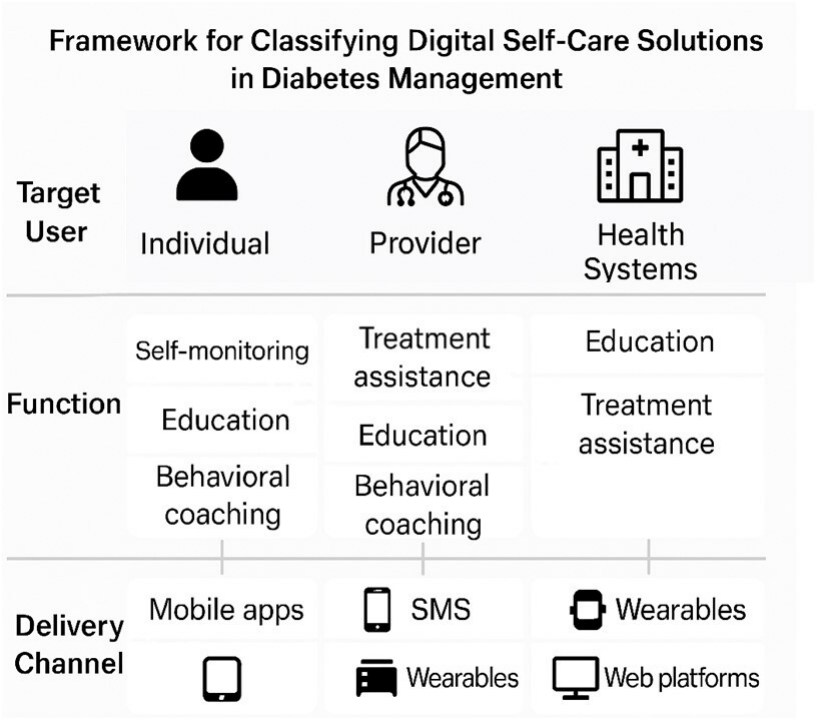
Adapted the WHO Self-Care Intervention Framework, illustrating how digital health solutions support diabetes self-management across three levels of intervention.

The inclusion criteria are as follows:

- Studies reporting on digital self-care systems and solutions for diabetes management and/or monitoring (eg, diet, physical activity, blood glucose control, treatment adherence, complication prevention and education). To ensure clarity and consistency, eligible digital solutions will be classified along three dimensions: (1) Function (eg, self-monitoring, behavioural coaching, education), (2) Delivery channel (eg, mobile apps, SMS, wearables, web platforms) and (3) Target user (individual, provider or system-level), in alignment with the WHO self-care intervention framework. Studies involving both adult and paediatric populations will be included. Where possible, data will be extracted and analysed by age group in order to explore age-specific trends in the use of digital self-care.^14^ This framework provides a structured basis for evaluating digital interventions across levels of healthcare engagement and ensures consistency in how self-care support is identified and analysed across diverse settings (figure 1). Included solutions must go beyond passive information delivery and demonstrate an active role in supporting diabetes self-management.

- Randomised and non-randomised controlled trials (RCTs), controlled before-after studies, interrupted time series studies, observational studies and case studies. Given that the review focuses on evaluating digital self-care interventions for diabetes management, appropriate comparators will be considered to contextualize the impact of these interventions including (1) Standard or usual care (conventional diabetes self-management approaches without the use of digital interventions, such as in-person consultations, printed educational materials and routine clinical follow-ups), (2) Non-digital self-care interventions (traditional self-care strategies that rely on manual tracking methods, paper-based logs or community-based support without technological integration), (3) Alternative digital solutions (comparison with other digital health technologies that may offer different features, levels of personalisation or engagement strategies) and (4) No intervention (situations where no structured self-care support is provided beyond general healthcare guidance).
- Studies evaluating commercially available products or prototypes (with real patients).
- Both published and unpublished studies.
- Studies involving adults and children with any type of diabetes, including type 1 diabetes, type 2 diabetes, gestational diabetes and other forms of diabetes, without restrictions on age or geographical context. Additionally, studies that include individuals with diabetes and other long-term conditions (eg, hypertension, cardiovascular disease, obesity or chronic kidney disease) will also be considered. The inclusion of these studies is important as multimorbidity is common among individuals with diabetes, and digital self-care interventions often target multiple health conditions simultaneously. Analysing such studies will provide a more comprehensive understanding of the effectiveness, feasibility and scalability of digital self-care solutions in real-world settings, where patients often manage multiple chronic conditions concurrently.
- Studies that examine social media as a complementary component within broader digital self-care interventions for diabetes management. For example, studies where social media is used alongside other digital health tools (such as mobile applications, telehealth services or wearable devices) could provide valuable insights into its role in enhancing patient engagement and adherence to self-care practices.

The exclusion criteria are as follows:

- Studies primarily addressing healthcare provider perspectives without direct involvement of individuals with diabetes.
- Opinion pieces, conference abstracts, book chapters, reports, dissertations or editorials without original research findings.
- Studies not available in English, French, Arabic, Portuguese, Spanish, Italian, Czech, Slovak and Chinese.
- Studies focusing exclusively on social media platforms for general health promotion, diabetes awareness or health communication without direct self-management interventions (eg, public health campaigns, influencer-driven awareness initiatives).

All identified citations will be uploaded into the Covidence systematic review software,^24^ and duplicates will be removed automatically.

Two reviewers (DEC and SA) will independently screen titles, abstracts and full texts to ensure eligibility. Any disagreements will be resolved by a third reviewer (AR).

### Stage 4: charting the data

A standardised data extraction form will be developed in Covidence, and pilot-tested to ensure consistency. Data extraction will be completed independently by two reviewers (DEC and SA). Extracted data will include the following entities:

- Study characteristics: author, year, country and study design. These elements are essential to contextualise the research within specific timeframes, geographic locations and methodological frameworks, providing insights into trends and potential biases.
- Participant characteristics: sample size, age, diabetes type and complications. Extracting these details will help assess the applicability of digital self-care interventions across diverse populations and identify variations in their effectiveness based on demographic and clinical factors.
- Digital self-care solution characteristics: type, brand, design, functionality and cost. We will extract specific AI-related features including, but not limited to, predictive analytics, personalised recommendations, decision support systems and automation of diabetes management tasks, machine learning applications (eg, algorithms used for glucose monitoring, insulin dose adjustments and lifestyle recommendations), integration with other digital tools, such as wearable devices, mobile applications and electronic health records. These aspects are crucial for understanding the scope of digital interventions, their accessibility and the features that contribute to effective diabetes management.
- Reported outcomes and usage statistics: key outcomes assessed in each study (eg, glycaemic control, treatment adherence, user satisfaction), and app usage metrics (eg, frequency of use, engagement levels, dropout rates) where available. This will help evaluate the effectiveness and real-world utilisation of digital self-care solutions.
- Barriers and facilitators: elements influencing the implementation and adoption of digital solutions, such as technological, financial and behavioural factors. This information will help identify critical challenges and enablers for the successful integration of digital self-care solutions in various healthcare settings.
- Ethical considerations: the integration of digital self-care solutions, including advanced technologies such as AI, raises several ethical considerations that must be carefully examined to ensure the responsible use of these interventions in diabetes management.^25^ This review will consider key ethical aspects including (1) Data Privacy and Security, (2) Informed Consent and User Autonomy, (3) Bias and Fairness in AI-based Interventions, (4) Commercial Interests and Conflicts of Interest, (5) Digital Divide and Accessibility and (6) Regulatory and Ethical Compliance. By addressing these ethical considerations, this scoping review aims to provide a comprehensive overview of how digital self-care solutions align with ethical best practices, identify potential risks and suggest strategies to enhance trust and transparency in their deployment for diabetes management.
- Equity considerations in access to digital self-care solutions: ensuring equitable access to digital self-care solutions for diabetes management is crucial, particularly for individuals in low-resource settings who may face significant barriers to adoption. The digital divide—characterised by disparities in access to technology, internet connectivity and digital literacy—poses a challenge to the widespread implementation of digital health interventions. To ensure a comprehensive assessment of equity in digital health interventions for diabetes management, we will extract variables aligned with the Digital Health Equity Framework^26^ including (1) Digital Determinants of Health (eg, access to technology, digital literacy, socioeconomic factors, health literacy), (2) Intervention Design and Implementation (eg, user-centred design, affordability, language and cultural relevance), (3) Outcomes Related to Equity (eg, disparities in health outcomes, engagement and retention rates) and (4) Contextual Factors (eg, policy Environment and healthcare infrastructure). Extracting these variables will enable a thorough evaluation of how digital health interventions address or perpetuate health inequities.

### Stage 5: collating, summarising and reporting the results

The results from the data extraction tool will be collected and summarised to form a comprehensive narrative review, including:

- Descriptive statistics and analysis of study and digital self-care solution characteristics.
- Thematic analysis of qualitative data to identify common themes and patterns.
- Comparative analysis to explore usability, safety and other outcomes across digital solutions.

The findings will be presented in a narrative format, supported by descriptive statistics, tables and figures to illustrate key points. The review will highlight trends, gaps in the literature and implications for future research and practice. Findings related to ethical and equity considerations will be thematically analysed using a deductive approach based on the WHO Self-Care Framework and the Digital Health Equity Framework. Patterns and gaps will be highlighted across settings and populations.

## Discussion

This scoping review will provide a comprehensive overview of the current landscape of digital self-care solutions and prototype systems for diabetes self-management. By systematically mapping the existing literature, this review will identify the types of digital tools and services available, their safety, usability and impact on self-management and patient-provider relationships. The findings will highlight trends, gaps and areas for future research, contributing to the development of more effective and user-friendly digital health interventions to improve diabetes self-management and support.

### Strengths and limitations

One strength of this scoping review is its rigorous and comprehensive search strategy, which includes multiple databases and grey literature, ensuring a broad capture of relevant studies. The use of a standardised data extraction form and independent screening by multiple reviewers will enhance the reliability and validity of the findings. Two independent trained extractors working in parallel will aim to enhance the reliability of the results by minimising individual biases, promoting consistency and reducing potential errors. To ensure comprehensive reporting of results, rigorous decision-making processes will be implemented to resolve discrepancies between extractors by a third extractor.

Moreover, the inclusion of diverse study designs (RCTs, observational studies, case studies and reviews) will provide a comprehensive view of the available evidence.

The chosen entities, such as participant characteristics, digital self-care solution characteristics, barriers and facilitators, are essential to understand the current research on digital tools for diabetes self-management, identify existing knowledge gaps and showcase effective practices. Including various study designs will ensure a well-rounded analysis that addresses the practical implications of our results and study quality. By aligning with the Digital Health Equity Framework,^26^ we can systematically identify barriers and facilitators to equitable access and propose strategies to enhance inclusivity in digital health solutions for diabetes management. This approach ensures that our review not only assesses the effectiveness of digital interventions but also their fairness and accessibility across diverse populations.

Although a formal risk of bias assessment will not be conducted, we will report any methodological limitations identified by the study authors, in order to provide context for the strength of the evidence base.

Another strength of the study is the multidisciplinary team with expertise essential for this broad scoping review. JC and ZP contribute clinical expertise in diabetes management and TPE, grounded in practice and research leadership within the WHO Collaborating Centre and international networks. GL and SJ, both based at the WHO, contribute expertise in global digital health policy and non-communicable disease management, ensuring alignment with international frameworks and priorities. CP and LVL bring expertise in digital health and AI. LVL also contributes health systems and workforce planning expertise through his role at the WHO Collaborating Centre on Health Workforce Policy and Planning. BP contributes expertise in therapeutic education with a broader European perspective through his leadership in the European Society for Therapeutic Education. AR provides methodological and health systems research expertise with a focus on digital innovation in population health. VDA, as an information specialist, is responsible for developing and optimising the search strategy, including database navigation and grey literature sourcing. DEC, SA and CFEK contribute to data screening and extraction and bring complementary perspectives from public health, nutrition and epidemiology. All reviewers involved in screening and data extraction have been trained in scoping review methodology.

However, certain limitations should be considered. The review will be limited to studies published in certain selected languages, potentially excluding relevant research conducted in other languages. The review may encounter difficulties in synthesising the findings due to the heterogeneity of the outcomes, digital tools and evaluation methodologies across the studies. Additionally, we chose to conduct our grey literature search exclusively through Google Scholar due to its broad indexing of academic and non-academic sources, including preprints, technical reports, policy documents and conference materials. This method is commonly used in scoping reviews to identify grey literature across diverse domains and was deemed appropriate given the wide scope of this review and the multidisciplinary nature of digital self-care solutions. However, we acknowledge that grey literature sources indexed in databases such as Embase and CINAHL were not included in our search strategy, which may limit our ability to capture all relevant non-journal literature. This decision was made to keep the scope manageable and transparent within available resources and aligns with the review’s aim to map existing published evidence rather than exhaustively document all grey sources.

Finally, the quality of the included studies will not be formally assessed, as this is not typically within the scope of a scoping review. This limitation could affect the strength of the conclusions regarding the efficacy and safety of the identified digital self-care solutions.

### Patient and public involvement

Although patients and the public were not involved in the design or conduct of this study, efforts will be made to ensure the findings are accessible to interest-holders. This will include healthcare providers, policymakers and patient advocacy groups. Dissemination strategies may involve creating summary reports, infographics and presentations tailored to different audiences to facilitate understanding and utilisation of the results.

### Ethics and dissemination

As this study involves the review of existing literature, ethical approval is not required. The findings will be disseminated through peer-reviewed publications, conference presentations and engagement with relevant interest holders to inform policy and practice. Additionally, the results will be shared with patient advocacy groups and other community organisations to support the broader adoption of digital self-care solutions in diabetes management.

## Supplementary material

10.1136/bmjopen-2025-100506 online supplemental file 1
