# Supplementary material for: Use of digital self-care solutions for diabetes long-term management: a scoping review protocol
Source: BMJ Open. 2025 Oct 29;15(10):e100506. doi: 10.1136/bmjopen-2025-100506 (PMC12574351; doi:10.1136/bmjopen-2025-100506)
Supplement: online supplemental file 1 [file bmjopen-15-10-s001.docx]

Table 1: Search terms used in Medline (PubMed interface, 1946 onwards)

|  |  | Concept | Search |
| --- | --- | --- | --- |
| Medline (PubMed) | # 1 | Diabetes mellitus | "Diabetes Mellitus"[Mesh] OR "Hyperglycemia"[Mesh] OR "blood sugar"[TIAB:~5] OR "insulin resistance"[TIAB:~5] OR diabete*[TIAB] OR diabetic*[TIAB] OR glucose[TIAB] OR glycaemi*[TIAB] OR glycemi*[TIAB] |
|  |  | AND |  |
|  | #2 | Digital heath technologies | "Digital Health"[Mesh] OR "Telemedicine"[Mesh] OR "Mobile Applications"[Mesh] OR "Artificial Intelligence"[Mesh] OR "Wearable Electronic Devices"[Mesh] OR "Internet-Based Intervention"[Mesh] OR "Remote Sensing Technology"[Mesh] OR "connected device*"[TIAB] OR "digital application*"[TIAB] OR "digital health"[TIAB:~5] OR "digital therapeutic*"[TIAB] OR "digital therapeutics"[TIAB:~5] OR "eHealth"[TIAB] OR "health apps"[TIAB:~5] OR "health technolog*"[TIAB] OR "mHealth"[TIAB] OR "mobile app*"[TIAB] OR "mobile health"[TIAB] OR "online intervention*"[TIAB] OR "smart device*"[TIAB] OR "smartphone app*"[TIAB] OR "telehealth"[TIAB] OR "telemedicine"[TIAB] OR "virtual health"[TIAB] OR "virtual reality"[TIAB:~5] OR "wearable*"[TIAB]) |
|  |  | AND |  |
|  | #3 | Self-care | "Blood Glucose Self-Monitoring"[Mesh] OR "Self Care"[Mesh] OR "Patient Education as Topic"[Mesh] OR "Health Education"[Mesh] OR "Disease Management"[Mesh] OR "Patient Compliance"[Mesh] OR "glucose monitoring"[TIAB:~5] OR "glycaemia control"[TIAB:~5] OR "glycaemia monitoring"[TIAB:~5] OR "glycemia control"[TIAB:~5] OR "glycemia monitoring"[TIAB:~5] OR "glycemic control"[TIAB:~5] OR "health education"[TIAB:~5] OR "lifestyle management"[TIAB:~5] OR "Patient adherence" [TIAB] OR "Patient cooperation" [TIAB] OR "patient education"[TIAB:~5] OR "patient empowerment"[TIAB:~5] OR "remote monitoring"[TIAB:~5] OR "self care"[TIAB:~5] OR "self management"[TIAB:~5] OR "self management”[Mesh] OR "self monitoring"[TIAB:~5] OR "self-efficacy"[TIAB:~5] OR "selfmanagement"[TIAB] OR "selfmonitoring"[TIAB] OR "telemonitoring"[TIAB] OR "Therapeutic complian*" [TIAB] OR "therapeutic education"[TIAB:~5] OR "Treatment complian*" [TIAB] |

-------------------------------------
