# Supplementary material for: Use of digital self-care solutions for diabetes long-term management: a scoping review protocol
Source: BMJ Open. 2025 Oct 29;15(10):e100506. doi: 10.1136/bmjopen-2025-100506 (PMC12574351; doi:10.1136/bmjopen-2025-100506)
Supplement: Supplementary file 2 [file bmjopen-2025-100506.reviewer_comments.pdf]

PEER REVIEW HISTORY

BMJ Open publishes all reviews undertaken for accepted manuscripts. Reviewers are asked to complete a checklist review form and are provided with free text boxes to elaborate on their assessment. These free text comments are reproduced below.

ARTICLE DETAILS

Title (Provisional)

Use of Digital Self-Care solutions for diabetes long-term management: a Scoping review protocol

Authors

Correia, Jorge; Fakih El Khoury, Cosette; El Chaar, Dayana; Arakelyan, Stella; Rasooly, Alon; Loffreda, Giulia; Joshi, Surabhi; Cohen, Jean-David; De Andrade, Vincent; Benoît, PÉTRE; Lapão, Luis Velez; Perrin, Caroline; Pataky, Zoltan

---

VERSION 1 - REVIEW

**Reviewer**	**1**
**Name**	**Smaldone, Arlene**
**Affiliation**	**Columbia University**
**Date**	**04-Apr-2025**
**COI**	**None**

Thank you for the opportunity to review the protocol for this scoping review. The development and testing of digital applications for diabetes self management is important and I think the questions posed for this scoping review are appropriate. However, what concerns me is that there have been a number of prior reviews, some quite recent, in this area. None were acknowledged in the introduction and that did bother me. How will your proposed review be different or add to this growing body of literature? Based on what has been accomplished in prior reviews, is a scoping review the appropriate review type?

The proposed team for this review is quite large yet in the protocol only three members have an identified role in reviewing title and abstract, full text and data charting. I would like to see the roles of the team defined - is there a methodologist? an informatics expert? a diabetes specialist? expertise in artificial intelligence methods? These are just examples of the expertise that the team should bring to this review.

Please include as either a table or figure your full search strategy including Boolean operators and MeSH terms for one of your planned database searches and describe how the strategy will be adapted for other search databases. How you plan to conduct a search for grey literature using Google Scholar should also be explicitly stated.

The WHO framework is referred to numerous times. Please include a figure that illustrates the WHO framework as it may have implications for how you will organize the synthesis of your results.

In addition to what you have already included, the data charting section should also include what was measured for each of these digital applications and did it improve by using the app.

I'm surprised that in the data charting section you have not included the testing of these apps -- what was measured and if and how it improved by using the app. Also, if use statistics are presented in the included studies, this would be valuable to your scoping review.

**Reviewer**	**2**
**Name**	**Chang, Ji Eun**
**Affiliation**	**New York University, Public Health**
**Date**	**15-May-2025**
**COI**	**None**

Thank you for this opportunity to review your article, "Use of Digital Self-Care solutions for diabetes long-term management: a Scoping review protocol".

This protocol addresses an important area in diabetes care—the role of digital self-care technologies in improving self-management. The protocol is clearly written with many strengths including the explicit attention to health equity and ethics. I have a few comments/suggestions to clarify the proposed protocol.

First, there is a mismatch between the aim of the study and the proposed data extraction plan. Specifically, the authors note in the introduction that there is a gap in synthesizing the evidence regarding the impact of digital health technologies on outcomes. The authors further explain that the results from the data extraction tool will be summarized to “Comparative analysis to explore usability, safety, and other outcomes across digital solutions.” Given these aims, the authors should consider including some measures of outcomes in the extraction process/plan.

Second, the search strategy is not transparent. The authors describe the strategy in broad conceptual terms but do not include specific terminology or even an appendix of preliminary terms. For transparency and replicability, the authors should consider sharing at least one full search string and/or a table of controlled vocabulary (i.e., MeSH) and free-text terms.

Third, and this is related to the point above, the authors propose to assess different digital tools and technologies but do not provide a clear classification or search for these tools. There are different ways of understanding what “digital self-help tools encompass. The

authors point to the WHO framework for self care but this framework is much broader than just digital tools. The authors should be clearer on what will or will not be included as part of a digital tool and how these tools will be classified. For example, will they be classified by the setting of the intervention? The function? Platform?

Finally, the authors do not present a contingency plan for excess search volume. Given the broad inclusion criteria, multilingual search, and 20 year time span, the authors should describe how they will manage/adjust will narrow the scope if the resulting search results in an unmanageably large number of articles.

VERSION 1 - AUTHOR RESPONSE

Comments	Response
**Reviewer 1**	
Concern about prior similar reviews; what is the added value of this one?	As noted above, we now compare our study with four recent reviews and specify how ours differs methodologically, conceptually, and in terms of scope. This is detailed in the final paragraph of the Introduction.
Request to define team roles and expertise.	Instead of a separate section, we incorporated this into the "Strengths and limitations of this study" section, highlighting our interdisciplinary team's clinical, digital health, AI, methodological, and information science expertise.
Include full search strategy and explain grey literature process.	We have included a complete PubMed search strategy in Supplementary File 1, and clarified in the Methods that we will screen the first 300 results in Google Scholar, following recommended practice for grey literature identification.
Include a visual of the WHO framework.	We have included a new figure (“Figure 1”) presenting an adapted version of the WHO Self-Care Intervention Framework, overlaid with examples of digital self-care solutions

	categorized by function, delivery channel, and target user.
Expand data charting to include what is measured and outcomes.	We added a new data extraction category in the "Charting the Data" section: *Reported Outcomes and Usage Statistics*, covering clinical, behavioral, and engagement outcomes.
**Reviewer 2**	
Mismatch between aim and data extraction – outcomes not clearly planned.	we added a new data charting dimension focused on outcomes and real-world usage metrics to align with our stated goals of synthesizing impact and usability evidence.
Lack of transparency in search terms and structure.	Full PubMed search strategy now provided in Supplementary File 1, with reference in the Methods.
Classification of digital tools not clearly defined.	We refined the Study Selection section to explain that interventions will be classified by Function, Delivery Channel, and Target User, aligned with the WHO framework. We also updated Figure 1 to reflect this tri-dimensional approach.
No contingency plan for excess volume of results.	We now state that if the search yields an unmanageable number of studies, we will prioritize by diabetes type, intervention complexity, or study design, while documenting any exclusions transparently.

We appreciate your constructive feedback, which has significantly improved the clarity and depth of our manuscript. Thank you for your time and consideration.
